# Left ventricular remodeling after transcatheter aortic valve implantation (TAVI)

**DOI:** 10.1186/1532-429X-15-S1-E39

**Published:** 2013-01-30

**Authors:** Sebastian Gruenig, Florian Bönner, Yang Chul Boering, Marc W Merx, Tobias Zeus, Malte Kelm, Mirja Neizel, Burkhard Sievers

**Affiliations:** 1 Cardiology, University hospital Duesseldorf, Duesseldorf, Germany

## Background

Transcatheter aortic valve implantation (TAVI) offers a minimal invasive option for the treatment of patients with severe aortic stenosis at high risk for conventional surgery. The objective of this study was to investigate whether there is a positive effect on the left ventricular remodeling 6 month after transcatheter aortic valve implantation (TAVI) using cardiac magnetic resonance imaging (cMRI).

## Methods

20 patients with severe aortic stenosis (aortic valve area <1 cm^2^) underwent TAVI. 15 patients were conducted with a Core Valve® Transfemoral Bioprosthesis and 5 patients with an Edwards® Transapical Bioprosthesis. cMRI was performed on a 1.5 Tesla MR Scanner (Achieva, Philips, The Netherlands). Steady state free precession (SSFP) short axis (SA) cine imaging was determined for measurement of LV volumes (EDV,ESV,SV) and ejection Fraction (EF) before and 6 month after TAVI.

## Results

The average age of patients was 83.6 ± 10.5 years. At baseline, EF was 55±21%. Endsystolic volume (ESV) was 69±50 ml, enddiastolic volume (EDV) 140±46 ml and stroke volume (SV) 75±22 ml. 6 Month after TAVI there was a significant increase in EF (63±39%, p=0.003). EDV, ESV and SV did not change significantly at follow up (EDV 98±43 ml, p=0.8; ESV 61±39 ml, p=0.2; SV 78±20 ml, p=0.8). Furthermore, there was no remarkable difference in cardiac remodeling between Edwards® Transapical and Core Valve® Transfemoral bioprothesis in this small patient population.

## Conclusions

TAVI resulted in significant improvement of EF at follow-up in patients with severe aortic stenosis compared to baseline LVEF. Further studies with more patients have to be performed to investigate if there is a difference in cardiac remodeling after Edwards® transapical valve replacement and Core Valve® transfemoral valve replacement.

## Funding

None

